# A Novel Amperometric Biosensor Based on Poly(allylamine hydrochloride) for Determination of Ethanol in Beverages

**DOI:** 10.3390/s21196510

**Published:** 2021-09-29

**Authors:** Oana Maria Istrate, Lucian Rotariu, Camelia Bala

**Affiliations:** 1 LaborQ, University of Bucharest, 030018 Bucharest, Romania; oana-maria.dumitrascu@cdi.unibuc.ro (O.M.I.); lucian.rotariu@g.unibuc.ro (L.R.); 2 Department of Analytical Chemistry, University of Bucharest, 030018 Bucharest, Romania

**Keywords:** alcohol dehydrogenase, biosensor, screen-printed electrode, ethanol

## Abstract

Herein, we report on a new type of ethanol biosensor based on a screen-printed electrode modified with poly(allylamine hydrochloride). The alcohol dehydrogenase was immobilized on the surface of the sensor using the sol–gel matrix. Working parameters such as applied potential, pH, NAD^+^ concentration, storage conditions were optimized. A response range between 0.05 and 2 mM was found with a sensitivity of 13.45 ± 0.67 µA/mM·cm^2^ and a detection limit of 20 µM. The developed biosensor was used to detect ethanol in commercial beverages with good accuracy.

## 1. Introduction

Ethanol is one of the most important organic chemicals in human activities with a variety of uses in food industry, chemical synthesis, medicine and biotechnology. Ethanol can appear as a bi-product in various processes in the food industry and can reach harmful levels due to fermentation and distillation. It also causes alcohol poisoning in direct consumption by humans. For these reasons, ethanol detection is of great importance for clinical and industrial applications. Some of the most common detection methods for ethanol include high performance liquid chromatography [1], gas chromatography [2], capillary electrophoresis [3], colorimetry [4], Raman spectrometry [5], or tandem techniques [6,7]. Although some of the above methods are complex and reliable, they can have disadvantages such as the need to use previous processes of sample separation (distillation, pervaporation), can be time consuming, and instruments can be expensive and need trained operators.

The enzyme-based biosensors represent an attractive solution to overcome all these disadvantages. The use of biosensors for the quantitative analysis of ethanol offers several advantages, such as sensitivity, versatility, low cost and the possibility of miniaturization and integration into portable systems. Other advantages make reference to the fact that the biosensors do not require qualified personnel and laborious sample treatments. Enzymatic biosensors have evolved rapidly and are used successfully in clinical analysis [8], in the analysis of food [9] and alcoholic beverages [10]. The enzymes involved in the development of biosensors for ethanol detection are alcohol dehydrogenase (ADH) [11] and alcohol oxidase [12]. ADH is the most used in the development of enzymatic biosensors for ethanol detection, where the substrate detection is performed by measuring the amount of NADH formed in the enzymatic reaction.

One of the most important steps in the development of an electrochemical biosensor is the immobilization of the enzyme on the electrode surface. Literature reports different materials used for the immobilization of ADH, including cationic polymers [13], nanobiocomposites based on polyelectrolytes and carbon materials [14], graphene–Au nanorods hybrid nanosheets [15]. The use of these composite materials leads also to an enhancement of the electrochemical detection of NADH that finally improve the performances of the ethanol biosensors.

Polyelectrolytes are a wide class of compounds used as coating materials as well as immobilization matrices for some biomolecules in the development of biosensors [16,17]. The deposition of the polyelectrolyte layer on the electrode surface increases the active area of the electrode, and due to the electrostatic interaction between the analyte and the polyelectrolyte, leads to its accumulation on the electrode surface that can enhance the electrochemical detection of the analyte.

**Scheme 1:**
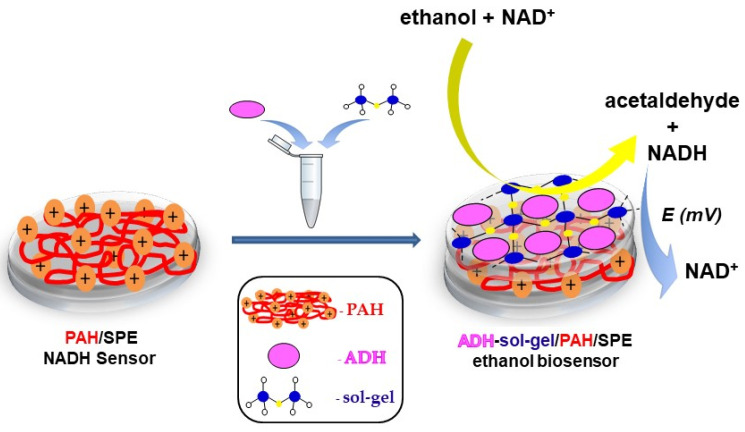
Schematic representation of the ethanol biosensor.

Herein, we report a new amperometric biosensor with immobilized alcohol dehydrogenase for the detection of ethanol in commercial beverages. Alcohol dehydrogenase was entrapped into a sol–gel matrix that was immobilized on the surface of screen-printed electrode (SPE) modified with poly(allylamine hydrochloride). The PAH/SPE enhances the accumulation of the NADH at the surface of the electrode and leads to an increase of the oxidation current, as was previously reported [18]. A schematic representation of the biosensor is shown in Scheme 1. The optimization of the working parameters and the characterization of the biosensor was performed by using amperometry.

## 2. Materials and Methods

### 2.1. Reagents

Alcohol dehydrogenase from braker’s yeast (ADH, 75,000 IU/g solid), nicotinamide adenine dinucleotide hydrate (NAD^+^), nicotinamide adenine dinucleotide reduced disodium salt (NADH), poly(allylamine hydrochloride) (PAH, MW 15 kDa), ethanol, ascorbic acid (AA), uric acid (UA), dopamine (DA), glucose (Glu) and acetaminophen (APAP), hydrochloric acid (HCl) were purchased from Sigma Aldrich and were used as received without further purification. Tetramethoxysilane (TMOS), methyltrimethoxysilane (MTMOS) and polyethylene glycol (PEG 600) were purchased from Fluka (Fluka Chemie GmbH, Buchs, Switzerland). All other chemicals were of analytical grade. Phosphate buffer (PBS) 0.1 M, pH = 8.8 was prepared from Na_2_HPO_4_ and NaH_2_PO_4_ and contains 0.1 M KCl. All solutions were prepared in ultrapure water (Millipore 18 MΩ∙cm).

### 2.2. Equipment and Materials

The Autolab PGSTAT 101 (Metrohm-Autolab B.V., Utrecht, The Netherlands) electrochemical workstation was used for cyclic voltammetry and amperometric measurements. Screen-printed electrodes (SPE) from Dropsens (Metrohm Dropsens, Oviedo, Spain) based on carbon working electrode; silver pseudo-reference electrode and carbon counter electrode (DRP-C110) were used for the preparation of the ADH biosensor. All potentials are reported vs. silver pseudo-reference electrode. All measurements were performed at room temperature. A magnetic stirrer was used to provide a constant convective transport during the amperometric measurements. The enzyme activity was determined spectrometrically using a Shimadzu UV-1650PC UV-VIS spectrophotometer (Shimadzu, Kyoto, Japan) by monitoring the NADH at 340 nm. Scanning electron microscopy (SEM) images were recorded with a Carl Zeiss AURIGA CrossBeam Workstation at an accelerating voltage of 2kV. pH measurements were performed with an InoLab WTW pH 730 pH-meter (Inolab WTW, Weilheim, Germany).

### 2.3. Preparation of PAH Modified Screen Printed Electrodes

Prior their modification, the screen-printed electrodes were pretreated using cyclic voltammetry by scanning the potential on the range −500 and + 500 mV in PBS (0.1 M, pH = 6) until reproducible voltammograms were obtained. This pretreatment, which leads to a negatively charged electrode surface, favors the formation and stabilization of the cationic polyelectrolyte film (PAH). The procedure has been previously published elsewhere [18]. After pretreatment, 5 µL aqueous PAH solution (0.5 mg/mL) was deposited on the surface of the working electrode and then the electrode was left to dry at room temperature for 24 h.

PAH modified SPE sensor was previously optimized and the reported results shown an enhanced electrochemical detection of NADH [18]. This sensor was used as NADH transducer for preparation of an ethanol biosensor based on immobilized ADH.

### 2.4. Preparation of ADH-Sol-Gel/PAH/SPE Biosensor

Alcohol dehydrogenase (ADH) was immobilized in a sol–gel matrix taking into account the simplicity and efficiency of the procedure that preserve the enzyme activity for a long period [19]. The enzyme activity of ADH was determined prior the immobilization. NADH produced in the enzyme reaction was detected by spectrometry at 340 nm [20]. The kinetic measurements allowed us to determine the enzyme reaction rate and express the real enzyme activity.

The immobilization matrix was prepared by mixing 5 µL of TMOS with 15 µL MTMOS, 40 µL HCl (20 mM), 44 µL ultrapure water and 4 µL PEG 600 [21]. The mixture was sonicated for about 15 min and then kept at 4 °C for about 6 h. After hydrolysis of the precursors and formation of the so-called “sol”, 10 µL of sol was mixed with 10 µL ADH (20 IU) and finally, 5 µL was dropped on the surface of working electrode modified with PAH film. Subsequently, the biosensor was left to dry for 24 h in desiccator at 4 °C in order to favor the condensation reaction and entrapment of the enzyme in polymer network of the sol–gel. The procedure was optimized in order to immobilize 5 IU of ADH on each PAH/SPE sensor.

## 3. Results

### 3.1. Morphological Characterization of the Electrodes

**Figure 1:**
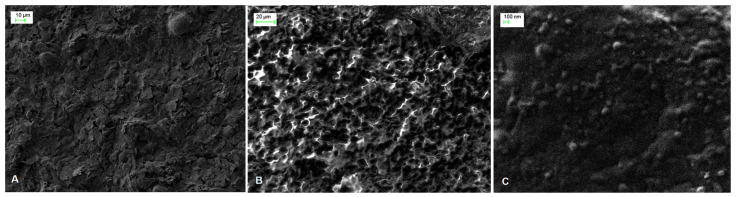
SEM images of (**A**) SPE, (**B**) PAH/SPE and (**C**) ADH-sol-gel/PAH/SPE sensors.

The surface morphology of SPE, PAH/SPE and ADH-sol-gel/PAH/SPE electrodes was studied by scanning electron microscopy (SEM). As one can see in the Figure 1A, the image of bare SPE electrode shows irregular flakes of graphite, which are randomly oriented. The SEM micrograph of the PAH film deposited onto the SPE electrode (Figure 1B) evidenced an ordered hemispherical morphology that provides a high active surface of the modified electrode. A smoother surface was observed after immobilization of the enzyme using sol–gel with small particles ascribed to the condensation process of “sol” in the presence of ADH (Figure 1C).

### 3.2. Optimization of Working Potential

**Figure 2:**
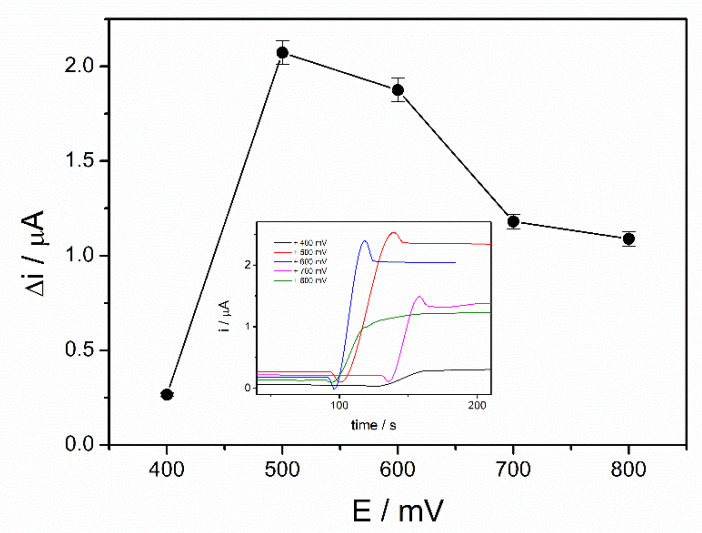
Influence of working potential on the biosensor response (PBS 0.1 M pH = 8.8, 10 mM NAD^+^, 4 mM ethanol, n = 3) (inset: chronoamperometric response curves at an applied potential between +400 and +800 mV).

The working potential influences the sensitivity of an amperometric biosensor and optimization of the applied potential leads to a significant improvement of the performances of the biosensor. The optimization of the working potential of ADH-sol-gel/PAH/SPE biosensor was achieved by chronoamperometric measurements in the presence of 4 mM ethanol and 10 mM NAD^+^ at applied potentials between +400 mV and +800 mV. The steady state currents before and after addition of ethanol were recorded. The variation of the current after addition of ethanol was graphically represented upon the applied potential as it can be seen in the Figure 2. A significant increase of the analytical signal can be observed from +400 mV to +500 mV, where it reaches a maximum value of 2.1 µA. At higher potentials, the current decreases and reaches approximately 1.2 µA at +800 mV. The potential of +500 mV was considered the optimal working potential and it was used further for all successive measurements.

### 3.3. Optimization of the Working pH

**Figure 3:**
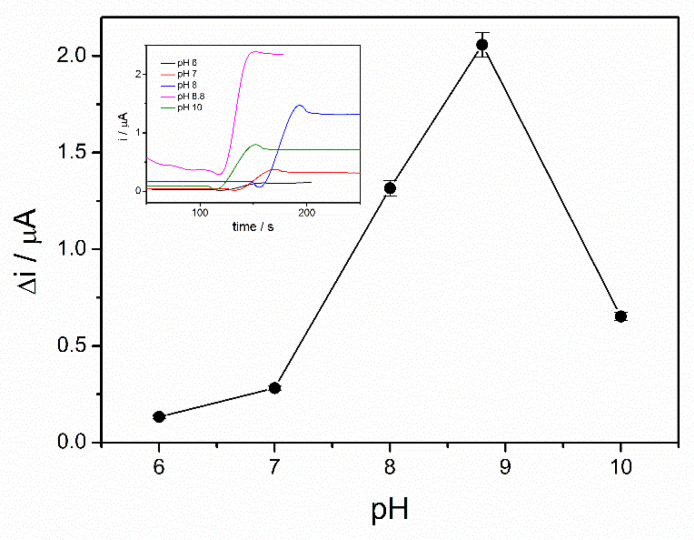
Influence of pH on the biosensor response (PBS 0.1 M, 10 mM NAD^+^, 4 mM ethanol, n = 3) (inset: chronoamperometric response curves at pH between 6 and 10).

Enzymatic oxidation of ethanol in the presence of ADH is dependent on the pH. Moreover, the enzyme immobilization method can affect the working pH of the ethanol biosensor. Therefore, in order to find the optimal working pH, the chronoamperograms were recorded in the presence of 4 mM ethanol and 10 mM NAD^+^ and the pH of PBS buffer 0.1 M was varied in the range from 6 to 10. A significant increase of the analytical signal was observed over the pH range of 6–8 with a maximum reached between 8 and 9, as can be seen in Figure 3. The NADH oxidation current decreases at higher pH, corresponding to a lower reaction rate. The optimal pH of alcohol dehydrogenase reported in the literature ranges between 8 and 9 [22]. An optimal pH of 8.8 for the immobilized ADH is in the above-mentioned range, which proves that the sol–gel matrix does not modify the enzyme properties. The immobilized enzyme seems to act similar to the native enzyme. As a hydrophilic matrix, the sol–gel preserves the enzyme activity and does not hinder the interaction with the substrate [19]. All subsequent studies were performed at pH of 8.8.

### 3.4. Optimization of the Coenzyme Concentration

**Figure 4:**
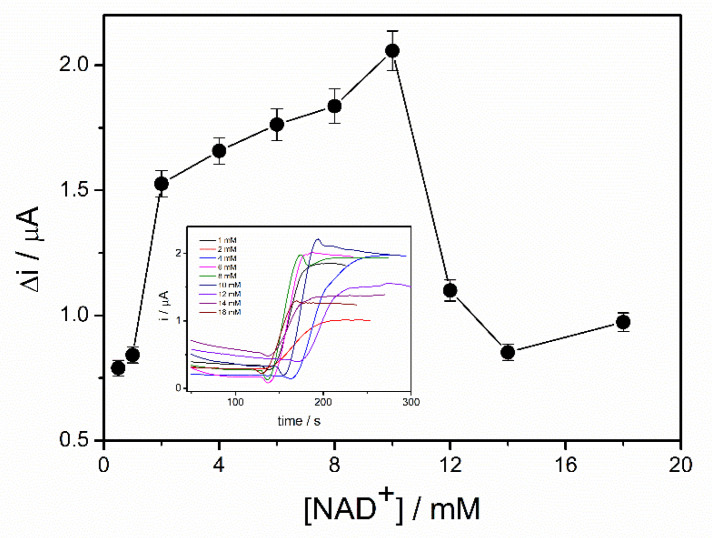
Influence of NAD^+^ concentration on the biosensor response (E = +500 mV, PBS 0.1 M pH = 8.8, 4 mM ethanol, n = 3) (inset: chronoamperometric response curves recorded for NAD^+^ concentrations range 0.5–18 mM).

The coenzyme (NAD^+^) plays an important role in the oxidation of the ethanol in the presence of ADH. The highest reaction rate and, consequently, the highest biosensor response can be achieved if an excess of NAD^+^ is provided. In order to study the influence of NAD^+^ concentration on the response of the ADH-sol-gel/PAH/SPE biosensor, chronoamperometric measurements were performed at an applied potential of +500 mV in 0.1 M PBS buffer, pH = 8.8, and an ethanol concentration of 4 mM. The concentration of NAD^+^ was varied between 0.5 and 18 mM. Figure 4, which exhibits the response of the ethanol biosensor in the presence of different concentration of NAD^+^, shows an increase of the oxidation current on the range 0.5–10 mM NAD^+^. The decrease of the signal at concentrations higher than 10 mM can be attributed to an electrode surface blocking effect. The diffusion issues inside the sol–gel matrix and the accumulation of the NAD^+^ at the surface of the electrode can explain this behavior.

Consequently, a concentration of 10 mM NAD^+^ was considered optimal and it was used for biosensor calibration and detection of ethanol in real samples.

### 3.5. Effect of the Rehydration on the Biosensor Response

The preparation of the biosensors implies the immobilization of ADH in the sol–gel matrix. During the formation of the gel matrix, the biosensors were kept in a desiccator, which leads to its partial drying. Before using the ethanol biosensor, it is recommended to perform a rehydration of the biosensor in order to enhance the permeability of sol–gel and increase of the diffusion rate of the compounds involved in the enzyme reaction at the level of the electrode surface [23].

**Figure 5:**
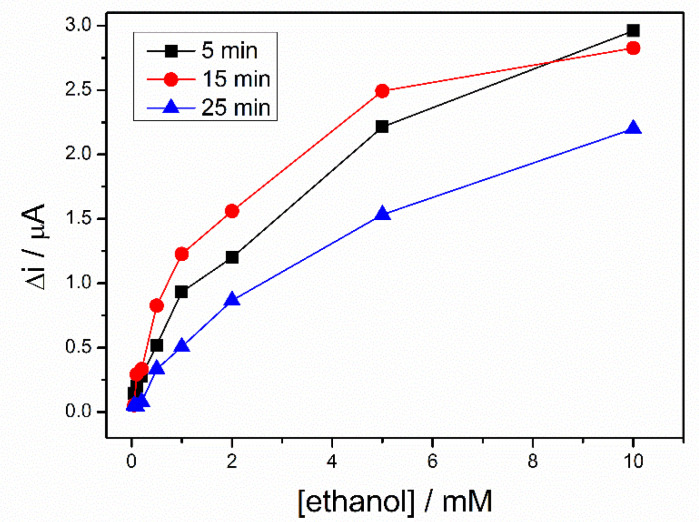
Calibration graph of ADH-sol-gel/PAH/SPE biosensors at different storage time in buffer solution, 5 min (black line), 15 min (red line), 25 min (blue line), (E = +500 mV, [NAD^+^] = 10 mM, PBS 0.1 M pH = 8.8).

To rehydrate the sol–gel matrix, three biosensors, prepared in the same conditions, were kept in PBS buffer for different time ranging from 5 to 25 min. Then, a calibration of each biosensor was performed in 0.1 M PBS (pH = 8.8), containing 10 mM NAD^+^. A difference of potential between working electrode and pseudo-reference electrode of +500 mV was applied and response of the biosensors were recorded to successive additions of ethanol. The Figure 5 shows the calibration curves of the three biosensors. For the whole range of ethanol concentrations, the highest signal was recorded after a hydration time of 15 min., this being considered the optimal hydration time used for all subsequent measurements.

### 3.6. Ethanol Biosensor Calibration

**Figure 6:**
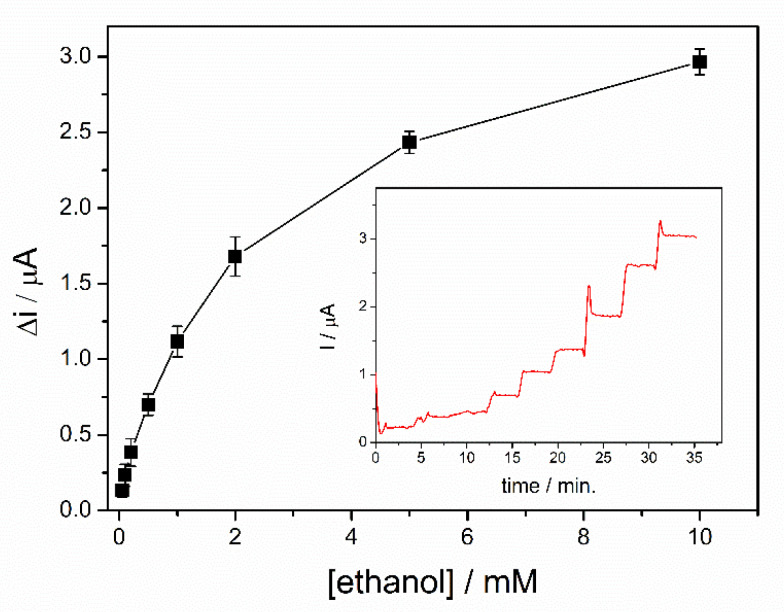
Dynamic response range of ADH-sol-gel/PAH/SPE biosensor (E = +500 mV, [NAD^+^] = 10 mM, PBS 0.1 M pH = 8.8, n = 3) (inset: typical biosensor response curve).

Calibration of the ethanol biosensors was realized in a 5 mL electrochemical cell under continuous stirring in the optimized condition. A potential of + 500 mV was applied after immersion of the biosensor in 5 mL of PBS buffer. After reaching the steady state signal, successive volumes of ethanol were added. A rapid stabilization of the analytical signal was observed after each addition, with a response time of 19s for the entire range of concentrations tested, calculated to reach 90% of the steady state current value. The calibration curve of the biosensor corresponding to the range of ethanol concentrations between 0.05–10 mM is shown in Figure 6, where each point represents the mean value for three measurements. For each ethanol concentration, the variation of the current was determined as the difference between the steady state current after the addition of ethanol and the steady state current for the buffer solution. Two linear domains were highlighted from the dynamic response range presented in the Figure 6. The first linear range corresponds to the domain between 50 and 200 µM (i (µA) = 0.052 + 1.692∙[ethanol](mM)), where a specific sensitivity of 13.45 ± 0.67 µA/mM·cm^2^ was determined and a detection limit of 20 µM was calculated for a signal-to-noise ratio of 3. A specific sensitivity of 5.74 ± 0.35 µA/mM·cm^2^ was calculated for the second linear range selected in the domain 0.2–2 mM (i(µA) = 0.313 + 0.721∙[ethanol](mM)). The calibration graph shows a saturation trend of the biosensor response at concentrations higher than 2 mM. Consequently, due to the low sensitivity of the biosensor in this range, the relevant response of the biosensor was limited to the range 50–2000 µM.

Table 1 presents the performances of ethanol biosensors based on polymer modified electrodes reported in the literature. Compared to other enzyme biosensors for ethanol, the ADH-sol-gel/PAH/SPE biosensor exhibits high sensitivity, low detection limit for a response range of approximately two decades.

### 3.7. Stability of the ADH-Sol-Gel/PAH/SPE Biosensor

**Figure 7:**
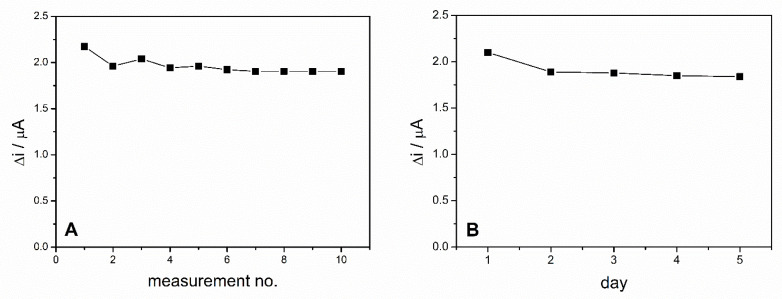
(**A**) Operational stability of the ADH-sol-gel/PAH/SPE biosensor. (**B**) Long term stability of the ADH-sol-gel/PAH/SPE biosensor E = +500 mV, PBS 0.1 M pH = 8.8, [NAD^+^] = 10 mM, ethanol 4 mM).

The operational stability of the ADH-sol-gel/PAH/SPE biosensor was studied by performing successive measurements of the same concentration of ethanol during the same day. These measurements were performed in 0.1 M PBS buffer, pH = 8.8 in the presence of 10 mM NAD^+^ and 4 mM ethanol every 2 min. Between measurements, the biosensor was stored in phosphate buffer solution. The biosensors response for 10 successive measurements is presented in Figure 7A. The relative standard deviation (RSD) was 4.4% for 10 measurements, which illustrates a very good operational stability of the ethanol biosensor.

The long-term stability of the biosensor was studied by chronoamperometric measurements performed on the same biosensor every day for 5 days. Before each measurement, the biosensor was kept for 15 min in PBS pH = 8.8 and when not in use, was stored in the desiccator at 4 °C. The biosensor exhibited a decrease of the response with 10% in the second day and then maintained relatively constant for the next days (Figure 7B).

### 3.8. Selectivity of the ADH-Sol-Gel/PAH/SPE Biosensor

The selectivity of the biosensor was evaluated in the presence of compounds such as ascorbic acid (AA), uric acid (UA), acetaminophen (APAP), dopamine (DA) and glucose (Glu) present in various real samples that are reported as potential interferents in the detection of ethanol. Amperometric measurements were performed in the optimized conditions for detection of ethanol, previously reported by successive addition of ethanol and interfering compound in a molar ratio of 1:1.

Table 2 shows the current ratios calculated as the report between the steady-state current after addition of interfering compounds in a solution of 1 mM ethanol and the current recorded in 1 mM ethanol before the addition of the interferent. As can be seen, only acetaminophen interferes with the detection of ethanol, leading to an increase of the signal with 18%. These results allowed us to conclude that the detection of ethanol with the developed biosensor can be performed without the occurrence of possible interferences for food product including alcoholic beverages.

### 3.9. Detection of Ethanol in Alcoholic and Non-Alcoholic Beverages Samples

The ADH-sol-gel/PAH/SPE biosensor was used to detect ethanol in commercial beer. One alcoholic and one non-alcoholic beer from the same trademark were used in this study and each sample was analyzed in triplicate. The ethanol content reported on the bottle label is 5% vol, which represents a molar concentration of 0.86 M. The beer sample was diluted 1:40 before measurements and 100 µL of the diluted sample was added in the 5 mL electrochemical cell, containing 0.1 M PBS pH = 8.8 and 10 mM NAD^+^.

Ethanol concentration was calculated using the second linear range of the biosensor for the alcoholic beer sample. The determined ethanol content was 0.93 ± 0.06 M yielding to a 108% recovery.

The currents recorded in the case of non-alcoholic beer sample were below the detection limit and addition of ethanol 1 mM leads to a recovery of 1.06 mM.

The ethanol concentration found in the alcoholic beer showed a good agreement with the ethanol content declared by the producer.

## 4. Conclusions

This study reports the development of a new enzyme biosensor based on alcohol dehydrogenase for the detection of ethanol in beverages. The electrochemical oxidation of NADH, performed at low potential with respect to a silver pseudo-reference electrode, is supported by coating the carbon screen printed electrode with a layer of poly(allylamine hydrochloride) that enhances the accumulation of the NADH at the surface of the modified electrode and leads to an increase of the oxidation current of this compound [18]. The enzyme was immobilized using a sol–gel matrix that preserves the enzyme activity and ensures fast diffusion of the coenzyme to the surface of the electrode. The main parameters, such as working potential and pH, NAD^+^ concentration, storage condition, were optimized. The ADH-sol-gel/PAH/SPE biosensor exhibits a large dynamic response range, fast response, high sensitivity, low detection limit with good stability and repeatability. Moreover, the biosensor proposed in this work present the advantage of being easy to fabricate, store and operate and would have great potential application in determination of ethanol in alcoholic beverages and food.

## Figures and Tables

**Table 1:** Comparison of the performances of some ethanol biosensors based on polymer modified electrodes.

Electrode Material	pH	Linear Range (µM)	LOD (µM)	Sensitivity (µA/mM·cm^2^)	RSD (%)	Ref.
MWCNTs-AuNPs-PNR	7.75	320.2–1000	96.1	3.9	1.57	[11]
RA	7.75	23.71–1000	7.1	10.83	1.65	[24]
MWCNTs-Polyarg	7.4	5–1000	0.65	21.04	5.5	[25]
PANI-PAAMPSA	8.5	5–2000	N.R.	5.19	N.R.	[26]
CNCs	7	~5000	300	0.15	7.8	[27]
TiO_2_/MWCNTs/Nafion/MB	7	50–1100	25	7.93	7.3	[28]
Fe_3_O_4_Au/MnO_2_	6.8	0.1–1 M	70	0.02	4.0	[29]
MB	8	50–15,000	80	0.94	3.8	[30]
PVI_13_dmeOs-PEGDGE	6	1–250	1	3.36	N.R.	[31]
PAH	8.8	50–200 200–2000	20	13.45 5.74	4.36	This work

MWCNTs: multiwall carbon nanotube, AuNPs: gold nanoparticles, PNR: poly neutral red, RA: rosmarinic acid, Polyarg: polyarginine, PANI: polyaniline, PAAMPSA: poly(2acrylamido-2-methyl-1-propane, CNCs: carbon nanocages, TiO_2_: titania sol gel, MB: Meldola’s Blue, Fe_3_O_4_Au: gols magnetic nanoparticles, MnO_2_: manganese dioxide, PVI_13_dmeOs: [Os(4,4-dimethylbipyridine)2Cl_2_] +/2+ and poly(1-vinylimidazole), PEGDGE: poly ethylene glycol diglycidyl ether, N.R.: not reported.

**Table 2:** Influence of interfering compounds on the detection of ethanol.

Interfering Compound	Current Ratio ^1^ E:I = 1:1
Glucose	1.0
Uric acid	1.0
Dopamine	0.9
Ascorbic acid	0.95
Acetaminophen	1.18

^1^ Current ratio = I_E+I_/I_E_ where I_E+I_ = response of 1 mM ethanol (E) in presence of interfering compounds (I) and I_E_ = response of 1 mM ethanol in the absence of interfering compounds.
